# Resilience of and recovery strategies for weighted networks

**DOI:** 10.1371/journal.pone.0203894

**Published:** 2018-09-11

**Authors:** Xing Pan, Huixiong Wang

**Affiliations:** School of Reliability and Systems Engineering, Beihang University, Beijing, China; Universidad Nacional de Mar del Plata, ARGENTINA

## Abstract

The robustness and resilience of complex networks have been widely studied and discussed in both research and industry because today, the diversity of system components and the complexity of the connection between units are increasingly influencing the reliability of complex systems. Previous studies have focused on node failure in networks, proposing several performance indicators. However, a single performance indicator cannot comprehensively measure all the performance aspects; thus, the selected performance indicators and recovery strategies lack consistency with respect to the targeted complex systems. This paper introduces a novel stress–strength-balanced weighted network model based on two network transmission hypotheses. Then, with respect to different concerns of the complex network, we propose two modified network performance measurement indicators and compare these indicators in terms of their trends in the attack process. In addition, we introduce several network recovery strategies and compare their efficiencies. Our findings are as follows: (1) The evaluation and judgment of the network performance depend on the performance measurement indicators we use. (2) Different recovery strategies exhibit distinct efficiencies in recovering different aspects of network performance, and no strategy exists that can improve all the network performance aspects simultaneously. (3) The timing of the recovery is proved to have a deep influence on the cost and efficiency of network recovery; thus, the optimal recovery strategy for a damaged network varies with the extent of the damage. From the results of the simulation of the attack-recovery process, we conclude that while defining and analyzing complex network models, we should adjust our network topology, weight assignment, and performance indicators in accordance with the focal characteristics of complex systems so that we can use the network model to build robust complex systems and efficient logistics and maintenance strategies.

## Introduction

Since the end of the 20th century, the notion of a network has attracted huge attention in scientific research. Network models can be studied as an abstraction and simplification of real-life complex systems, e.g., electrical power grids, the Internet, transportation networks, biological networks, and other self-organizing systems [1,2]. The nodes of a network represent the units or hubs of the real system, and the edges represent the paths carrying substances, information, energy, service, or even failure. However, in network models, the diversity of the internal properties or the individual behavior of nodes are simplified or even neglected. Such simplification ignores some specific attributes of the real systems but provides us with an approach to study the joint effects of large quantities of components that constitute a large system.

The last decade has witnessed an intensive study of the robustness and resilience of complex networks, and the study has been boosted by a series of discoveries about the dynamic characteristics of networks, including cascading failure [3,4], phase transition, percolation [5], and synchronization [6,7]. In the last few years, several studies have investigated the network response to node failures, i.e., the network robustness to node removal [8]. However, in real-life systems, edge failure is also an important factor responsible for performance degradation. In evolving systems, e.g., power grids and the Internet, the overload of network components is a major cause of failure [9,10]. An overloaded sensitive edge of a power grid can lead to capacity loss in the edge, and an overloaded edge in the central flow of the network can result in cascading breakdowns [11]. In communication networks, damage to optical fiber cables can partially overload data delivery, resulting in a regional interruption of Internet services [12]. In biological neural networks, edge failure such as the malfunction of neural conduction can cause a local or systemic paralysis. Cats et al. [13] developed a public transport robustness assessment model that evaluates the performance of a transportation network by computing edge criticality and affirmed the importance of considering edge capacity reduction in the optimization of public transportation systems. As the scale of the model network increases, the effect of edge failure on network robustness becomes greater and the neglect of edge attributes will generate one-sided conclusions.

To investigate network performance, scientists have proposed different performance measurement indicators based on network topological characteristics. The performance of a network can involve different aspects, and some classic tools have been developed to investigate these aspects, e.g., the size of the largest connected component (the number of nodes present in the giant cluster) and the characteristic path length (the average topological distance between a pair of connected nodes) [14]. In network science, whether network performance can be determined by a given set of measurements is termed as network observability [15]. With regard to the vulnerability of network topology, Shunkun et al. [16] investigated the network observability and proposed efficient network augmentations based on their findings. Boas et al. [17] carried out a detailed analysis on the sensitivity of different network performance measurements, compared the stability and discriminability of the measurements, and found effective performance indicators for different types of networks. Nogal et al. [18] built a performance indicator that accounts for recovery patterns under disruptive events and examined its validity using a dynamic restricted equilibrium model. Sun and Zeng [19] proposed a local betweenness-based performance measurement indicator and applied it to analyze their hybrid recovery method. Their results corroborated the advantages of their recovery strategy for public transportation.

In the last few years, the resilience of a network has also been widely discussed in both research and industry. Resilience is the ability of a network to bounce back to a desired performance level after facing malicious attacks or random failures [20,21]. Vlacheas et al. [22] conceptualized the idea of network resilience and validated an end-to-end resilience ontology by categorizing the exchanged information into Profiles, Context, and Policies. Previous studies [23–25] have proposed a comprehensive methodology for topology generation, and the analytical and experimental techniques used for evaluating the network attributes depend on the efficiency of the recovery strategy, i.e., the reconnection sequence. The efficiency of a recovery strategy indicates how much network performance can be recovered in a given number of edges or nodes once they are reconnected. Di Muro et al. [26] proposed a recovery strategy to repair the nodes in the mutual boundary of functional clusters in two interdependent networks based on a critical probability of recovery above which the system is restored and below which it collapses. The recovery process of a network is analogous to the establishing process of a new network, but a partially destroyed network retains some microscopic topological characteristics of the initial network, which affect the efficiency of the recovery [19]. Additionally, the timing of recovery, i.e., the point of intersection of the attack and recovery processes, affects the efficiency of the recovery. Costa [27] compared the efficiencies of different approaches for network augmentation, and the results indicated that the initial growth scheme has little effect on the network resilience enhancement and that different augmentation schemes vary in terms of the efficiency of recovery.

In this study, we first develop different types of edge attack strategies and recovery strategies, using which we compute different sequences for edge removal and reconnection. Then, we simulate the process of performance degradation and performance recovery of a model network according to the developed attack strategies and recovery strategies. By comparing the trends of network performance under different combinations of attack strategies, recovery strategies, and recovery timing, we study the robustness and resilience of a complex network and thereby propose a series of rules for network fault prevention.

## Model and methods

In this section, we introduce the model network, attack strategies, recovery strategies, and network performance measurement indicators.

### Stress–strength-balanced Barabási–Albert network model

**Fig 1:**
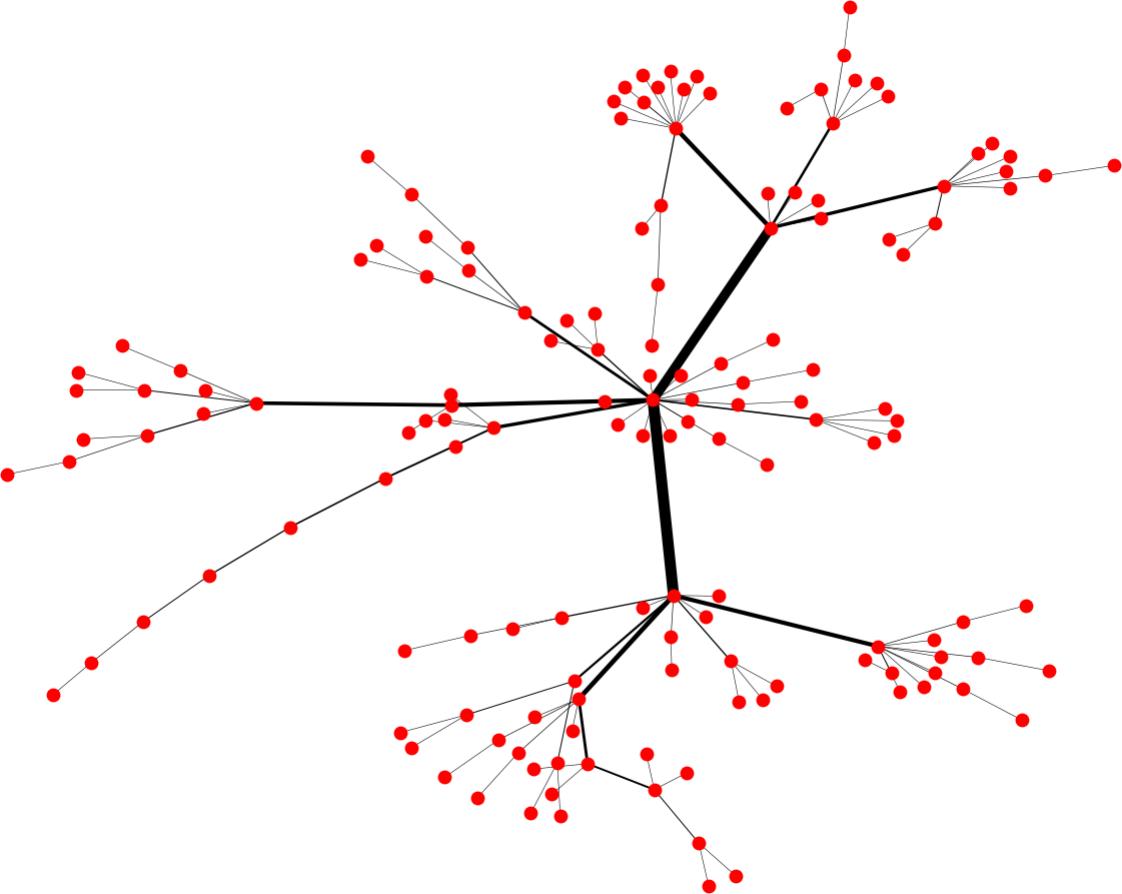
Stress–strength-balanced Barabási–Albert network consisting of 150 nodes and 149 edges.

We propose a stress–strength-balanced network model on the basis of the Barabási–Albert (BA) network model to study the robustness and resilience of scale-free networks. This 500-node, 996-edge model network is used in the simulation and analysis, described in the following section. A smaller model network is shown as an example in Fig 1. The thickness of the edge represents the weight associated with it, and the weight reflects the capacity of each edge.

To construct the stress–strength-balanced model, we developed two basic hypotheses:

1. Transmission hypothesis: the model network is designed to transmit information or substances between nodes, such that a fixed and equal quantity of information or substances is transmitted between all pairs of nodes.
2. Stress–strength-balanced hypothesis: the network is designed properly and the initial configuration of a stress–strength-balanced network perfectly satisfies the transmitting demand of the network.

In this manner, the stress and strength (or capacity) should be balanced in the initial configuration of the network. To accord with the above hypotheses, we assign the edge capacity, i.e., the weight of each edge, as a linear normalization of the edge betweenness centrality before edge removal.

First, we adopt the topology of the BA network model in this research. The connectivity of the BA network follows a scale-free power-law distribution, i.e., *P*(*k*)*~k*^*-γ*^, which is in accordance with the growth and preferential attachment features of many real-life complex systems [28]. We generate a BA network consisting of 500 nodes and 996 edges; in this network, the nodes and edges are added to the network sequentially and stochastically according to the connectivity algorithm of the BA network model.

Then, we add stress–strength-balanced weights to the edges based on the stress of each edge. The specific steps are as follows:

- According to the above transmission hypothesis, the main function of the model network is to transmit information or substances between nodes and that an equal quantity of information or substances is transmitted between each pair of nodes. The weight of each edge indicates the transmission capacity of the edge.
- Based on the hypothesis above, we compute the stress of each edge by calculating the edge betweenness centrality, which is the number of shortest paths from all nodes to others that pass through that edge [29]. The higher the betweenness centrality of the edge, the higher the stress acting on the edge.
- Based on the stress–strength-balanced hypothesis, the initial configuration of the network is supposed to perfectly satisfy the transmitting demand of the network; i.e., the capacity of each edge is designed to be adaptive to the quantity of information or substance flow. Hence, we assign the weight of each edge to be the linear normalization of the edge betweenness centrality. This can be mathematically represented as follows:
  $$w_{i}=\frac{b_{0i}}{\max\left(b_{01},b_{02},\ldots b_{0n}\right)}$$ (1)
  where *b*_*0i*_ represents the initial betweenness centrality of edge *i* and *w*_*i*_ represents the weight associated with edge *i*.

Before the attack, the stress and capacity of a network are balanced. Once the weight of each edge is assigned (i.e., once the capacity is assigned), it will not change (unless the edge is removed) during the evolution of the network. As the edges are removed, however, the structure and topology of the network change, thereby influencing the route of the transmission flow. This will destabilize the stress of each edge, induce component failure, and eventually break the balance of the load distribution, degrading network performance.

### Network performance measurement

We use two network performance measurement indicators described in the literature and propose connection rate and stress–strength index as novel performance indexes based on the stress–strength model network.

#### LCC size

The largest connected component (LCC) size, i.e., the size of the largest connected subgraph in the network, is frequently used as a measure of network performance [2,8,14,28]. In a real-life network, the LCC can reflect how many nodes or members in a network can transmit substances, information, or energy or provide services to each other; however, the weakness of the LCC measure is that it neglects the remaining subgraphs and components of the network. In addition to the nodes in the LCC, some groups of locally connected nodes can function in a degraded mode as inferior communities [30–32]. Based on this concern, we propose a new measurement, connection rate, which reflects the situation not only in the largest component, but also in other smaller subgraphs.

#### Connection rate

We measure the network performance by calculating the proportion of the connected pairs of nodes. Note that a connected pair of nodes means there is a path (a single edge or a series of edges and nodes) between them. Statistically, connection rate is a function of the sizes of each subgraph in a network. Therefore, when using connection rate to calculate the network performance, all the subgraphs are concerned, which overcomes the shortcoming of LCC size. The connection rate can be mathematically represented as follows:

$$Cr=\frac{2n_{cp}}{N(N-1)}\times 100\%$$ (2)

where *n_cp_* represents the number of connected node pairs and *N*(*N–* 1)/2 is the number of all node couples in the network. For a globally coupled network, the connection rate *Cr* = 1.

Based on our hypothesis that an equal quantity of substances or information is transmitted between each pair of nodes, as long as there is a path between two nodes, the nodes can sustain the transmission between them irrespective of the component the nodes belong to. In this manner, the connection rate can reflect the network performance, considering both the large components and inferior communities.

#### Weighted efficiency (EFF)

We use a network performance measurement indicator presented by Bellingeri and Cassi [8], which accounts for edge weights. In binary networks where each pair of nodes can only be either connected or disconnected, the shortest path is the minimum number of edges needed to travel from one node to another. Relatively, in weighted networks, the shortest path is the minimum sum of weights needed to travel between a pair of nodes. In a network that weight represents the transmission capacity, higher weight implies “wider and faster routes”, so we take the reciprocal of the weight of each edge to calculate the shortest weighted path. The minimum lengths of shortest weighted paths between all pairs of nodes constitute a square matrix, and the weighted efficiency (EFF) is the mean of the values in this square matrix. A detailed description can be found in Bellingeri and Cassi’s paper [8]. Note that the weighted efficiency (EFF) is a performance measurement, which is distinct from the term “efficiency of attack/recovery strategy”. In the following sections, we use the abbreviation “EFF” to avoid confusion with the term “efficiency of attack/recovery strategy”.

Our study reveals a weakness of EFF as a measurement indicator. By assessing the minimum weighted path between the nodes, EFF shows the connectivity of the network but neglects the balance between the stress and strength of the network. An edge becomes overloaded when the stress applied to it exceeds its capacity. From the perspective of failure mechanics, overloading an isolated device can lead to component performance degradation or failure [33], whereas in a network an overloaded edge can result in the loss of capacity, ultimately triggering a cascading failure [11]. In existing network performance measurements, the transmission between nodes is implemented through a path that has the shortest weighted length. In this case, if an edge has a very high transmission capacity, the reciprocal of its weight will be low, and consequently it might be frequently chosen as the shortest path to transmit and might end up suffering from overstress. However, the EFF measurement cannot reflect the overload in a network.

**Fig 2:**
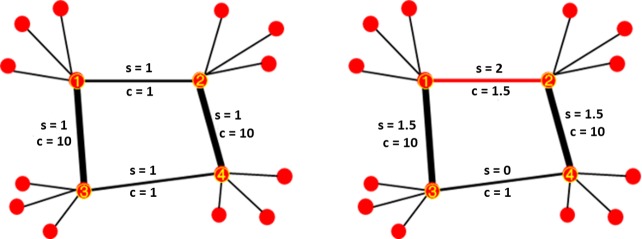
Example of overload phenomenon. “s” represents the stress applied to the edge, and “c” represents the strength of the edge.

An example of this phenomenon is shown in Fig 2. Fig 2 shows two networks, in which the capacity (c) of edges is artificially assigned, and the stress (s) is calculated through its betweenness centrality to simulate the phenomenon of load balancing. As shown in the network on the left in Fig 2, when we assign an equal capacity to edge (1,2) and edge (3,4), the load of the network is balanced. The network shown on the right in Fig 2 is identical, except that the capacity of edge (1,2) is artificially increased from 1 to 1.5. As a consequence of this alteration, edge (1,2) lies in the shortest weighted path between nodes 3↔4, 1↔4, and 2↔3. Worse still, the EFF of the network increases from 0.3313 to 0.3491, which represents a 5.4% increase in network performance. However, this change to a single edge induces 50% more stress on edges (1,3) and (2,4) and a 33% overload on edge (1,2) (s > c). Therefore, an increase to the weight of a pivotal edge in a network can increase the stress of a network and break the balance of the transmission, but the EFF algorithm does not reflect this degradation in performance.

#### Stress–strength index

We propose a stress–strength index as a supplement to the above measurement:

- Stress: Calculate the edge betweenness centrality as described above. A higher value of the edge betweenness centrality indicates that an edge is chosen more number of times to be in the shortest weighted path and that a greater amount of substances and information is transmitted through this edge, which implies that more stress is exerted on this edge. We used the ratio of the current value of the edge betweenness centrality to its initial value before edge removal. The stress of edges can be mathematically represented as follows:
  $$s_{ji}=\frac{b_{ji}}{b_{0i}}$$ (3)
  where *s*_*ji*_ represents the stress of edge *i* after the attack or recovery step *j*, and *b*_*ji*_ represents the betweenness centrality of edge *i* after the attack or recovery step *j*.
- Strength: The strength is the transmission capacity of each edge, derived by Eq (1), in the initial configuration of the network. As the attack process begins, the strength of the edges does not change.
- Stress–Strength Index: The ratio of the strength to the stress of the edge reflects the load condition of each edge. We define the stress–strength index as the average ratio of the strength to the stress of all edges. A higher stress–strength index implies that the edges (i.e., the transmission system) are working under lower loads compared to their capacity. This can be mathematically represented as follows:
  $$\eta_{j}=\frac{\sum_{i=1}^{n}\frac{w_{i}}{s_{ji}}}{n}$$ (4)
  where *w*_*i*_ represents the strength of edge *i* derived by Eq (1), *s*_*ji*_ represents the stress of edge *i* in the attack or recovery step *j*, and *η*_*j*_ represents the stress–strength index in the attack or recovery step *j*.
  In the simulation of the example shown in Fig 2, the stress–strength index decreases from 0.1363 to 0.0889, which indicates that network load balancing has degraded by 34.8% after changing the weight of a single edge. Therefore, the stress-strength index reflects network overload.

The stress–strength index is not concerned with how many nodes or system members are served. Rather, it calculates the average ratio of edge capacity to stress, and hence is a direct reflection of the network load and redundancy. Relatively, the connection rate focuses more on the proportion of the nodes that can transmit substances or information between them, but it does not account for the operating condition. Different network performance measurement indicators actually emphasize different aspects of the network performance.

### Attack strategies

We use the following attack strategies:

#### Highest weight

Edges are removed according to the weight. In each attack step, we remove the edge that is assigned the highest weight. Because the weights of the edges are assigned to reflect the betweenness centrality, in this attack strategy, the edges are also removed according to the betweenness centrality.

#### Random removal

Edges are removed randomly. In each step, we randomly choose an edge and remove it, which is consistent with the situation in the random failure stage in a product life cycle.

### Recovery strategies

Inspired by the node attack strategies found in the literature, we use the following recovery strategies:

#### Subgraph

La Rocca et al. [34] studied the efficiency of reconnecting the subgraphs to the LCC based on a probability measure that renders the network more resilient to failure. Correspondingly, our strategy involves reconnecting nodes according to the size of the subgraphs of the network; i.e., larger separated connected clusters are preferentially reconnected to the LCC. Without loss of generality, we randomly choose a node from each selected subgraph for reconnection.

#### Degree

Nodes are reconnected according to the degree. If two nodes together have the largest sum of degree but they are not neighbors, they are connected first [35].

#### Path shorten

Nodes are reconnected according to the weighted distance between them. If the weighted shortest path between a pair of nodes is the longest among all pairs of nodes, we connect this pair of nodes first. As described above, the weighted shortest path is the minimum sum of weights needed to travel between a pair of nodes.

#### Hub to farthest

In a network, nodes that possess a high degree are termed as hub nodes. In this recovery strategy, we preferentially choose a hub node and connect it to a node that is farthest from it.

#### Random

In each recovery step, we randomly choose two nodes that are not neighbors and connect them. We use this random recovery strategy for comparison with the abovementioned targeted recovery strategies.

In all the abovementioned recovery strategies, the weights of the new edges are assigned to be the average weight of all the existing edges.

## Simulation and results

We simulate the attack-recovery process on the model network described above. In the simulation, we remove the edges from the network, recover the network, and measure the network performance during the process according to the attack strategies, recovery strategies, and performance measurement indicators introduced in the previous section. To eliminate the influence of randomness, trials that contain a random process are repeated three times and their results are averaged.

In this section, we (1) present the simulation results, (2) compare different network performance measurement indicators by analyzing their trends in the attack-recovery process, (3) compare the robustness of the network when edges are removed by different attack strategies, and (4) compare the efficiencies of different recovery strategies.

### Network performance measurement fluctuation in attack process

In the simulation of the attack process, we sequentially remove edges from the model network according to the attack strategies. By the end of the simulation, all the edges (996) are removed. During the attack process, we observe the trend of the network performance by computing the given measurement indicators. As introduced in the previous section, we have two attack strategies and four network performance measurement indicators. Our results and findings are as follows.

**Fig 3:**
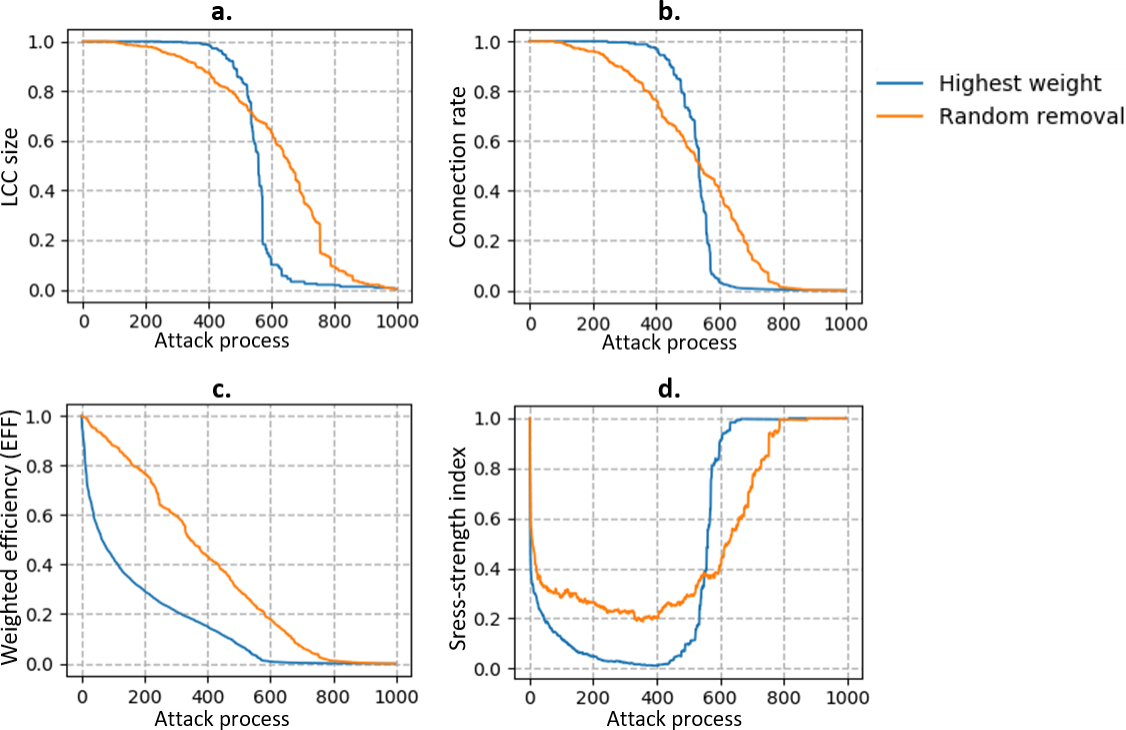
Trends of four performance measurement indicators in attack process. The horizontal coordinates represent the steps of the attack process, i.e., the number of edges that are sequentially removed from the network. The vertical coordinates represent the performance measurement indicators.

The graphs shown in Fig 3 indicate that different attack strategies exhibit different efficiencies. The measurement indicators in the two graphs on the left are derived from the literature, whereas in the right graphs, we use modified measurement indicators to observe the features that are considered in this study. From the trends of the LCC size and connection rate, we can see that the efficiency of the highest-weight strategy is less than that of the random-removal strategy at the early stage of attack. In the random-removal strategy, both the LCC size and connection rate start to decline from step 50, whereas in the highest-weight strategy, these two measurement indicators do not decline until step 300. When the performance starts to decline, the decline is rapid for the highest-weight strategy, and the performance of this strategy declines to zero earlier than that of the random-removal strategy. This phenomenon is called bond percolation in statistical physics [36–38], and percolation becomes more obvious when we use the weighted-removal strategy. In terms of EFF, the highest-weight strategy declines more sharply from the beginning and reaches zero earlier (step 600) than the random-removal strategy (step 800). In the highest-weight strategy, the stress–strength index decreases to zero, whereas in the random-removal strategy, the stress–strength index does not decrease to zero. In addition, after the network is completely destroyed, the stress–strength index can bounce back faster in the highest-weight strategy than in the random-removal strategy. Overall, the highest-weight strategy is more efficient in destroying the connection of the network; however, its efficiency has a lag when we observe the connection scale of the network.

In this study, we develop two modified measurement indicators—connection rate and stress–strength index—as supplements to the two original measurement indicators from the literature. The overall trend of the connection rate in the attack process is similar to that of the LCC size in our model and simulation, but the connection rate reaches zero earlier, which implies that the connection rate is more sensitive to edge failure than the LCC size. Another observation made in the simulation is that the stress–strength index shows a down–up trend, which indicates that the load of the network increases under early phase of failure, but as more edges are removed, the load of the network decreases. This is because when we remove a large proportion of the edges from the network (40% in our model), the network breaks down to pieces and most pairs of nodes are disconnected, resulting in lower edge loads and lower stress–strength index values. However, indicators other than the stress–strength index decreased monotonically. Consequently, these indicators fail to reflect overload in a network component.

### Comparison of different recovery strategies in recovery process

**Fig 4:**
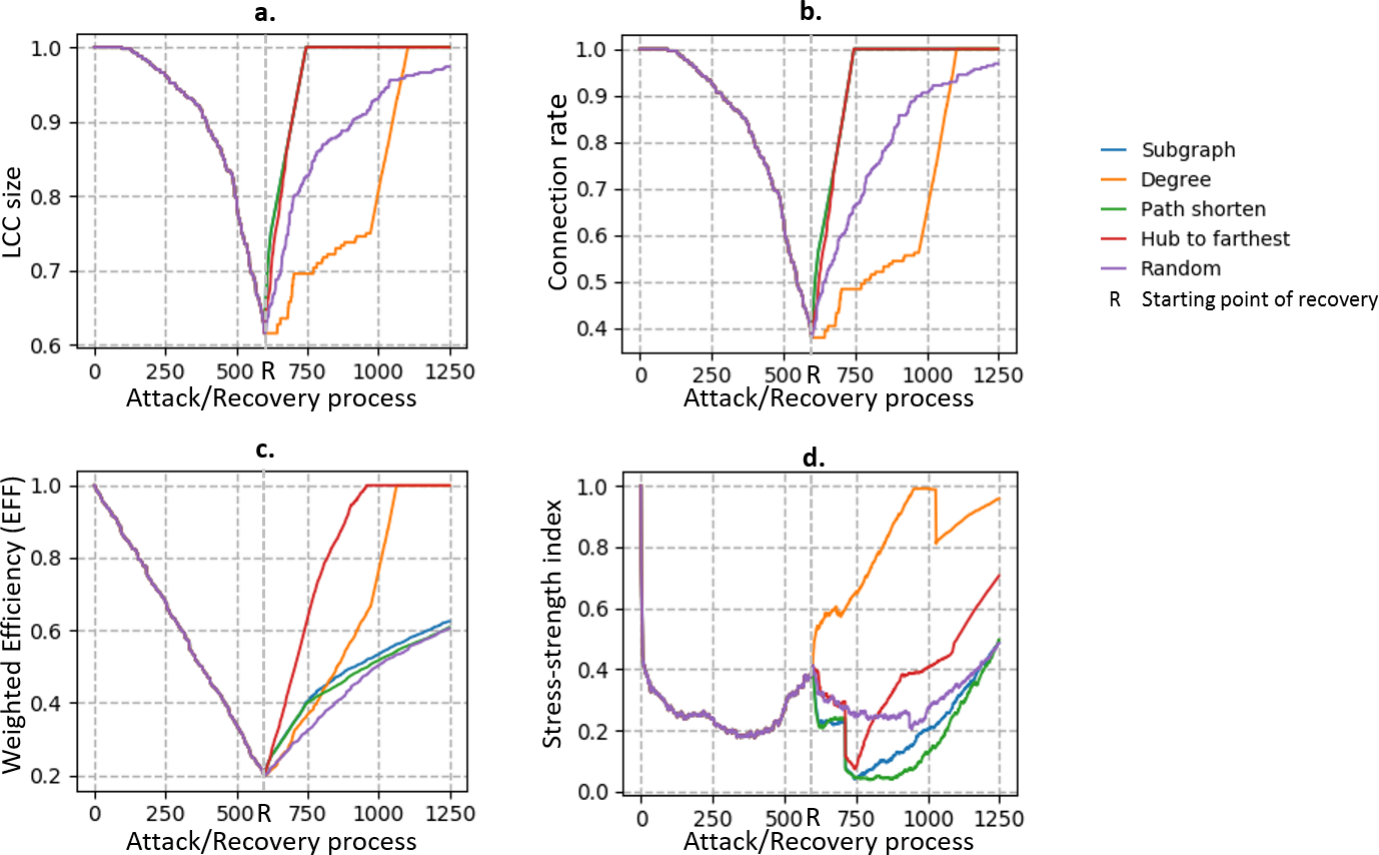
Trends of performance measurement indicators in attack and recovery processes. The horizontal coordinates are the steps of the process, and the vertical coordinates are the four performance measurement indicators described in the previous section. The mark “R” shows the starting point of the recovery process.

**Fig 5:**
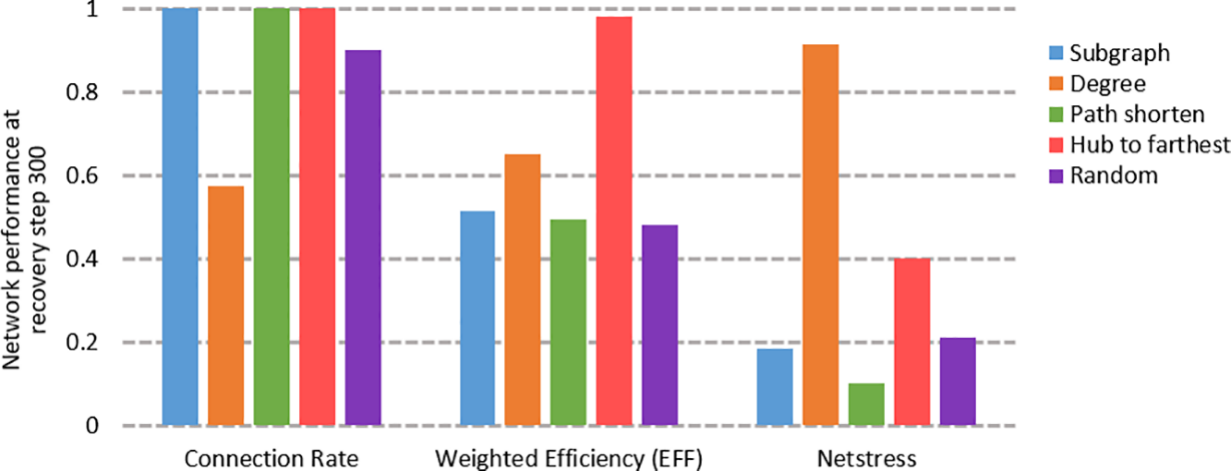
Network performance under different recovery strategies at recovery step 300.

In the simulation of the recovery process, we first break the network down by randomly removing edges from the network (random removal). When 60% of the edges (600 edges) are removed, we stop the removal and begin to the recover the network. In each recovery step, we add one edge to the network according to the recovery strategies. In both the attack and recovery processes, we trace the network performance by computing the performance measurement indicators in each step. We use the five recovery strategies and compare their efficiencies by observing the trend of the performance measurement indicators. The results are shown in Figs 4 and 5.

The efficiency of the recovery process varies with the recovery strategies we use. When we simply consider the connection scale of the network, i.e., the number of nodes that are connected to each other, we use the LCC size (Fig 4A) and connection rate (Fig 4B) as the measurement indicators of the network performance, and we find that subgraph and path shorten are the optimal recovery strategies. However, the hub-to-farthest strategy is the most efficient when we expect the paths between nodes to be averagely short and use EFF (Fig 4C) as the measurement indicator. When we consider the balance between the stress and strength of the edges, we use the stress–strength index as the measurement indicator, and the degree recovery strategy is the fastest in recovering the load condition of the network. Fig 5 shows the efficiency of different strategies at recovery step 300. Table 1 illustrates the optimal recovery strategy for each performance measurement indicator.

**Table 1:** Optimal recovery strategy for different performance measurement indicators.

Network Performance measurement	LCC size	Connection rate	Weighted efficiency (EFF)	Stress–strength index
Definition of measurement	Number of nodes in giant cluster	Probability of pair of nodes being connected	Average of minimum weighted path (reciprocal of weights) between all pairs of nodes	Proportion of overload edges in network
Description of measurement	Connection scale in giant cluster	Connection scale in all components of network	Average length of paths between nodes	Balance between stress and strength of all edges
Optimal recovery strategy	Subgraph	Subgraph	Hub to farthest	Degree
Steps needed to recover performance to initial state	149 (24%)	148 (23.9%)	343 (55.3%)	620 (100%)

Note that when EFF and Stress–strength index are used as the measurement indicators (Fig 4C and 4D), the performance trends of the random attack strategy and random recovery strategy (purple curves) are not symmetrical. This is because in the attack process, the weights of the removed edges are arranged such that the stress and strength are balanced, whereas in the recovery process, the weights of newly added edges are assigned at random. However, this kind of asymmetry does not exist for the LCC size and connection rate because they do not account for the weight of the edges. Another observed phenomenon is that the stress–strength index does not increase monotonically during the recovery process; instead, it shows fluctuation. This is because some isolated subgraphs in the network are reconnected to each other in the recovery process, thus connecting large quantities of node pairs in one step and increasing the loads of the edges in the network.

### Comparison of different timings of recovery process

**Fig 6:**
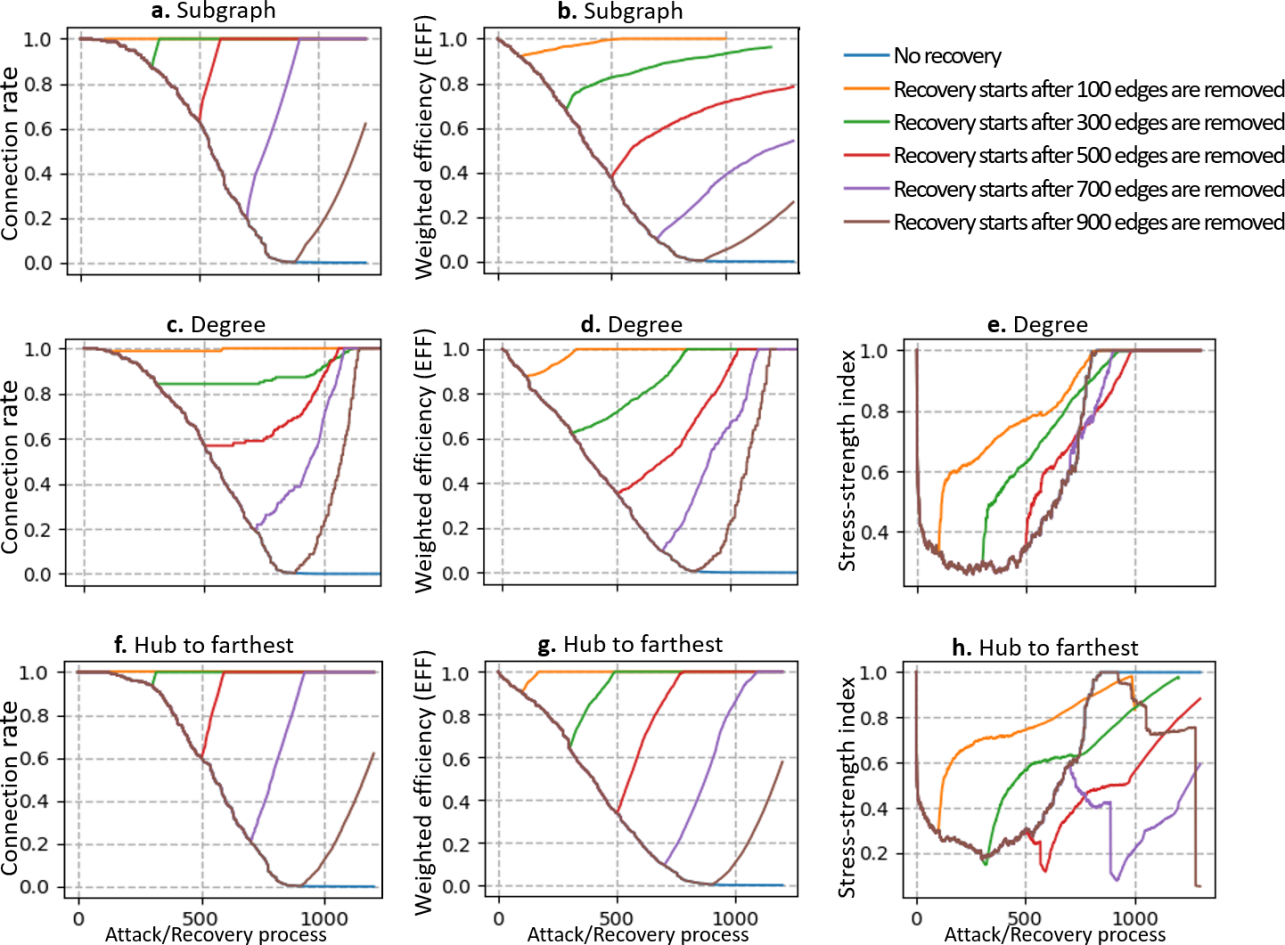
Trends of network performance indicators under different timings of recovery. Each row of graphs represents a recovery strategy, and each column of graphs represents a network performance measurement indicator. Different colors are used to represent curves with different timings of recovery.

**Fig 7:**
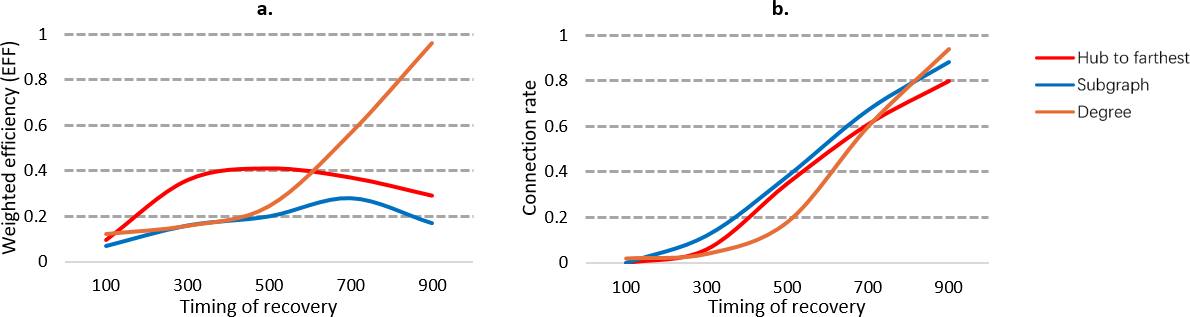
Comparison of recovery efficiencies under different timings. The horizontal coordinates represent the timing of recovery, i.e., the number of removed edges at the beginning of the recovery process. The vertical coordinates represent the performance recovery rate during the first 300 steps of recovery for each timing.

To analyze how the timing of recovery influences the efficiency of network recovery, we simulate the attack-recovery process at different timings by beginning the recovery at attack steps 100, 300, 500, 700, and 900 relatively. We select degree, hub to farthest, and subgraph as the recovery strategies and select the connection rate, weighted efficiency (EFF), and stress–strength index as the network performance measurement indicators. The trends of the measurement indicators under different combinations of timings and recovery strategies are shown in Figs 6 and 7. To control the uncertainty of the random attack process, all the recovery timings are simulated on a single attack process; thus, the attack sections for all the curves in each graph coincide with each other.

The above results indicate that the timing of recovery can influence the efficiency of recovery and that different strategies are more or less efficient at network recovery. In the degree strategy (Fig 6C, 6D and 6E), deeper damage to the network does not result in a longer recovery process. Instead, as more edges are removed from the network, the process of the recovery of the network performance to the initial stage becomes shorter, especially when we consider the connection rate of the network (Fig 6C). However, it should be clarified that the process to ensure the complete recovery of the network performance cannot be immoderately short; therefore, the curves will eventually become parallel to each other if we cause deeper damage to the network.

However, similar phenomena do not occur in the hub to farthest or subgraph recovery strategies (Fig 6A, 6B, 6F and 6G). In these two strategies, as we delay the timing of recovery, the slopes of the recovery curves become slightly smaller, which implies that more recovery steps are required to completely restore the network to the initial stage. As mentioned above, the subgraph strategy exhibits better efficiency in recovering the connection rate than the other strategies when the recovery is started at attack step 600. However, the above results indicate that the degree strategy is more efficient than the subgraph strategy when the network is deeply damaged (900 edges removed). This result indicates that the subgraph strategy is not the optimal recovery strategy for every degree of network damage.

Another observed phenomenon is that in the degree strategy, the stress–strength index (Fig 6E) of the network always increases monotonically at any degree of damage, whereas in the hub-to-farthest strategy, the stress–strength index (Fig 6H) exhibits a distinct decrease at the beginning if the recovery starts after half of the edges (500) are removed. This is because the network breaks into pieces when more edges are removed and the recovery process reconnects these isolated pieces, thereby rapidly increasing the transmission flow and edge stress. However, in the degree recovery strategy, the nodes with high degrees are reconnected first and the effect of load sharing surpasses the effect of an increase in the transmission flow; thus, the stress–strength index increases monotonically.

In Fig 7, we compare the recovery efficiencies of different recovery strategies under different timings. As the timing of the recovery process is delayed, the recovery efficiency of the degree strategy increases parabolically, but the efficiency of the subgraph and hub-to-farthest strategies fluctuates. This implies that the degree strategy is more suitable for recovering deeply destroyed (60% removed edges) networks.

From the above analysis, we can conclude that the timing of recovery can influence the efficiency of the recovery process. Overall, when the network is under slight edge failure, the hub-to-farthest strategy can restore the network performance fast; however, if most of the edges in the network (more than 80%) are removed, the degree strategy can provide the maximum recovery efficiency.

## Discussion

In this paper, we propose a stress–strength-balanced BA network model, which corresponds to real-life complex networks in terms of their growth and self-adaptive organizing characteristics. In the simulation and evaluation of the attack strategies, the neglect of the edge weight of complex networks will produce misleading network performance results [8]. Our study on the weight assignment algorithm shows the advantage of the stress–strength-balanced weighted network model over the randomly weighted ones found in the literature [17]. The effect of the edge assignment algorithm on the network performance and its influence on the attack and recovery strategies will be considered in future research.

We find that our analysis and judgment of the performance of complex networks depend on the measurement indicators we use. Different measurement indicators represent different aspects of the network performance. As a result, the optimal recovery strategies for different performance measurement indicators are different. A reliable performance measurement indicator should be adjustable in accordance with the characteristics of a complex system rather than providing narrow network performance just for simplification.

The results of our simulation of the network recovery process indicate that no recovery strategy exists that can be the fastest at simultaneously recovering all aspects of the network performance. The results in Figs 4 and 5 indicate that different strategies are more or less efficient at recovering network performance based on different indicators. Furthermore, the timing of recovery is also an influential factor in network recovery, and no recovery strategy exists that can provide the fastest improvement in performance at any degree of network damage. Our findings can be used to formulate better maintenance and logistics strategies for real-life complex systems. For complex systems in which the edge capacity can be considered to be infinite, e.g., a sea transportation network, the subgraph is the optimal recovery strategy; for systems in which the edge overload can cause severe component failures, e.g., power grids, the degree strategy can be applied to schedule an optimized maintenance strategy that accounts for the loads of edges. By applying the proposed network performance measurement indicators and recovery strategies, future research may examine whether these methodologies can be extended to other types of complex systems and whether these results can be replicated in other systems.

When comparing the results derived from different network models, we find that some simplifications of real systems produce one-sided results. For decades, the simplification and abstraction of complex networks has provided insight into the collective effect of network components [14,39]. However, our research results reveal that the inordinate simplification of networks will produce differentiated judgments of the network performance and will inadvertently give us impractical suggestions on recovering the complex systems from structural failures.

This study will open practical and straightforward extensions. First, to develop a practical network model and an optimal recovery strategy for a given system, it is important to recognize the focal and influential characteristics of the system and adjust both the model network and the performance measurement indicators in accordance with these characteristics. The topology and the weight assignment algorithm of the model network are desirable and realizable elements to adjust. Second, we can test the quality of the performance measurement indicators by comparing their trends in both attack and recovery process. When examining network performance, we should select the indicators that accord with our concerns or make appropriate modifications to them. Further, the timing of recovery is also an influential factor in the resilience process of weighted networks. We can maximize the efficiency of maintenance and logistics strategies for a complex system by optimizing their timings.
